# Cerebral amyloid angiopathy-related inflammation with posterior reversible encephalopathy syndrome-like presentation: a case report

**DOI:** 10.1186/s12883-022-02979-6

**Published:** 2022-12-03

**Authors:** Cheng Xia, Yan Lv

**Affiliations:** Department of Neurology, General Hospital of Northern Theater Command, Shenyang, China

**Keywords:** Cerebral amyloid angiopathy-related inflammation, Posterior reversible encephalopathy syndrome, Differential diagnosis, Vasogenic edema, Corticosteroid

## Abstract

**Background:**

Cerebral amyloid angiopathy-related inflammation (CAA-RI), which presents with acute or subacute cognitive or functional decline, focal or multifocal neurologic deficits, new onset of seizures, or a combination of seizures and neurologic deficits, shares clinical and radiologic similarities with posterior reversible encephalopathy syndrome (PRES). Differential diagnosis is critical because the treatment principle for these 2 conditions differs greatly. Here, we present a case of PRES-like CAA-RI and the strategy used to discriminate between the 2 conditions.

**Case presentation:**

A patient with probable CAA-RI was first thought to suffer from PRES. Initial high-dose methylprednisolone therapy caused rapid improvement of the neurologic symptoms but abrupt discontinuation of corticosteroids resulted in clinical relapse and deterioration. Subsequent reinitiation of high-dose methylprednisolone followed by tapering off of oral prednisone led to clinical and radiologic recovery at the 3-month follow-up.

**Conclusions:**

We suggest that in cases where it is difficult to distinguish between CAA-RI and PRES solely based on magnetic resonance imaging, a good response to corticosteroids and an apolipoprotein E (*ApoE*) ε4/ε4 genotype are critical for establishing a diagnosis of CAA-RI. If there is clinical deterioration, sudden withdrawal of high-dose corticosteroid during the active phase of CAA-RI should be avoided.

**Supplementary Information:**

The online version contains supplementary material available at 10.1186/s12883-022-02979-6.

## Background

Cerebral amyloid angiopathy-related inflammation (CAA-RI) was previously classified as a rare autoimmune encephalopathy subtype of cerebral amyloid angiopathy (CAA) that presents with acute or subacute cognitive or functional decline, focal or multifocal neurologic deficits, new onset of seizures, or a combination of seizures and neurologic deficits. These neurologic symptoms are correlated with white matter vasogenic edema, cerebral microbleeds (CMBs), and cortical superficial siderosis [1]. However, current evidence indicates that CAA-RI is an autoimmune encephalopathy linked to increased anti-amyloid beta (Aβ) antibodies in the cerebrospinal fluid (CSF) of patients with cerebral amyloid deposition, which is not strictly and solely related to a subtype CAA but also to Alzheimer disease, with and without confirmed CAA co-pathology [2]. A diagnosis of definite CAA-RI requires histopathologic confirmation; however, recent clinico-radiologic criteria [3] and detection of anti-Aβ autoantibodies in CSF [4] have improved the diagnostic capacity and obviated the need for an invasive brain biopsy.

Posterior reversible encephalopathy syndrome (PRES) is a rare clinico-radiologic syndrome typically presenting with acute or subacute altered mental status, headaches, seizures, and visual disturbances [5, 6]. White matter vasogenic edema is the main radiologic manifestation. At present, there are no universally accepted criteria for diagnosis, which is made based on clinical context and the clinician’s judgment [7]. The disorder is usually reversible when the precipitating cause (eg, hypertension) is treated with specific medication. Although corticosteroid therapy is not a standard treatment for PRES, it is helpful for relieving vasogenic edema caused by inflammation or trauma.

Here we describe the process of diagnosing and treating a case of PRES-like CAA-RI.

## Case presentation

**Fig. 1:**
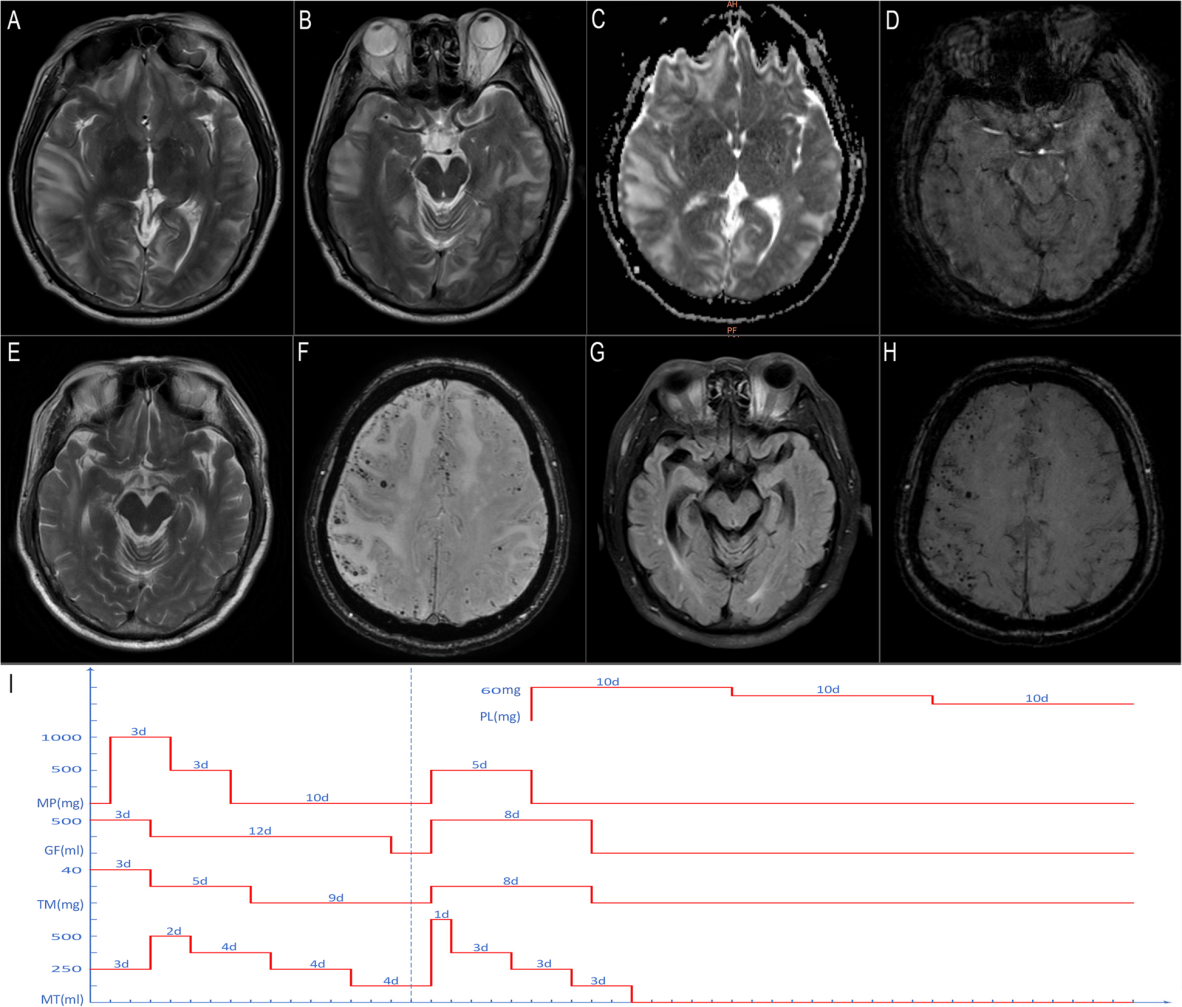
MRI findings and treatment timeline of the patient. **A–D** On day 2 after admission, the T2-weighted image showed diffused subcortical white matter hyperintensities in both hemispheres, predominantly in the temporal and occipital lobes (**A, B**); the apparent diffusion coefficient map showed increased diffusivity of the lesion consistent with vasogenic edema (**C**); and SWI showed several cortical and subcortical CMBs in bilateral temporal lobes (**D**). Following initial MRI, high-dose methylprednisolone (1000 mg/day for 3 days and 500 mg/day for 3 days) and dehydration therapy were administered, with rapid improvement of neurologic symptoms observed within 1 week. Head CT on day 10 after withdrawal of methylprednisolone revealed significant aggravation of brain edema (see supplementary Fig. 1). **E, F** Ten days after high-dose methylprednisolone reinitiation, MRI showed that the edema had largely resolved, but innumerable cortical CMBs appeared on SWI. **G, H** MRI at the 3-month follow-up showed remarkable regression of white matter hyperintensities, with no change in CMBs. **I** Treatment timeline showing course of the disease with different treatments. GF, glycerol fructose; MP, methylprednisolone; MT, mannitol; PS, prednisone; TM, torasemide

A 58-year-old man with a medical history of hypertension for about 30 years and ultrasonic lithotripsy 9 days prior presented to the emergency department with drowsiness, loss of appetite, emesis, and left limb weakness that had lasted 1 week. Physical examination revealed a blood pressure of 169/94 mmHg (range, 124–185 to 58–112 mmHg in the days prior to admission), lethargy, impaired memory and attention, and left hemiparesis. Head noncontrast computed tomography (CT) showed multiple subcortical hypodense changes. The next morning, the neurologic deficit was worsening, and there was also right limb weakness with bilateral extensor plantar response. At noon on the same day, the patient experienced a seizure. Emergency magnetic resonance imaging (MRI) showed diffuse hyperintensities on T2-weighted images localized subcortically in the white matter of both hemispheres, predominantly in the temporal and occipital lobes, and several small cortical and subcortical hypointense signal changes in bilateral temporal lobes consistent with CMBs on susceptibility-weighted imaging (SWI). The apparent diffusion coefficient map suggested vasogenic edema (Fig. 1. A–D). The angiogram was negative for cerebral venous sinus thrombosis.

Laboratory tests showed normal total and differential leukocyte counts, normal prothrombin and partial thromboplastin time, normal thyroid peroxidase antibody, Na 139 mmol/l, blood urea nitrogen 5.06 mmol/l, ammonia 28 μmol/l, and elevated serum C-reactive protein level (8.5 mg/dl; normal < 0.20 mg/dl). The CSF analysis showed 13 cells/mm^3^ (mononuclear cells, 10 cells/mm^3^; polynuclear cells, 3 cells/mm^3^) and elevated total protein concentration (207 mg/dl) with an opening pressure of 400 mmH_2_O. Both immunoglobulin G (IgG) and albumin were significantly increased in the CSF (464 mg/l and 1.57 g/l, respectively), suggesting severe disruption of the blood–brain barrier (BBB). The IgG index was mildly elevated (0.89; normal ≤ 0.70); however, no CSF oligoclonal IgG bands were detected. The workup for rheumatic disease was negative. CSF cultures showed no bacteria, mycobacteria, or fungi. Polymerase chain reaction was negative for herpes simplex virus and *Mycobacterium avium* complex. Serum and CSF antibodies suggestive of autoimmune encephalitis, central nervous system demyelinating disease, or paraneoplastic encephalitis were not detected.

A diagnosis of PRES due to shock wave lithotripsy and hypertension was initially suggested. Following MRI, the patient was immediately given intravenous (IV) methylprednisolone (1000 mg/day for 3 days and then 500 mg/day for 3 days), mannitol, glycerol fructose, and torasemide, with nicardipine by infusion for hypertension control; levetiracetam and valproic acid were also administered out of concern for ongoing seizure activity. The neurologic symptoms rapidly improved over the course of the following week, and the therapy was adjusted according the principle of PRES treatment with blood pressure control strictly maintained and IV mannitol tapered off.

However, the patient’s mental status again deteriorated, with several vomiting episodes on day 10 after withdrawal of methylprednisolone. The patient fell into a stupor with obtunded pupils. An emergency head CT scan showed patchy low-attenuation white matter lesions predominantly in the right hemisphere with distinct mass effect. (see supplementary Fig. 1).

The diagnosis of PRES was questioned at that time and CAA-RI was instead considered. Given that the worsening of brain edema may have been caused by methylprednisolone withdrawal, high-dose methylprednisolone (500 mg/day for 5 days) and dehydration therapy were immediately reinitiated followed by oral prednisone (initially 60 mg/day, followed by 5 mg tapering off every 10 days) (Fig. 1. I). Ten days after methylprednisolone reinitiation, MRI showed that the edema had subsided considerably although innumerable cortical CMBs appeared on SWI (Fig. 1. E, F). The patient’s mental status gradually improved and he was discharged 24 days later; the apolipoprotein E (*ApoE*) gene report revealed a ε4/ε4 genotype.

At the 3-month follow-up, the patient had achieved both clinical and radiologic recovery with oral prednisone 15 mg/day, although the multiple and scattered subcortical CMBs showed no change (Fig. 1. G, H). A final diagnosis of probable CAA-RI was made.

## Discussion and conclusions

The case presented here highlights the challenge of diagnosing a patient with a misleading initial presentation of CAA-RI. The medical history included hypertension and ultrasonic lithotripsy 2 days before the onset of subacute encephalopathy. The largely symmetric subcortical vasogenic edema in the brain MRI and acute occurrence of clinical symptoms rapidly resolved after high-dose corticosteroid, dehydration, and aggressive hypertension control. Such a course strongly suggested PRES, which was supported by the blood pressure on admission. PRES is seen in patients with a hypertensive emergency but is also observed with other conditions such as sepsis and eclampsia and exposure to immunosuppressive drugs. Ultrasonic lithotripsy has been reported as a cause of PRES [8]. The key pathophysiologic disturbance of PRES is endothelial dysfunction, which causes the breakdown of the BBB. Although in PRES, vasogenic edema is often seen bilaterally in parieto-occipital, holo-hemispheric watershed, or posterior frontal cortical and subcortical white matter on MRI [7], atypical distribution of vasogenic edema and CMBs have been reported [9, 10].

CAA-RI is increasingly considered as an autoimmune encephalopathy linked to increased anti-Aβ antibodies in the CSF of patients with cerebral amyloid deposition [4], which has also been reported in clinical trials of amyloid modification therapies [11]. Vasogenic edema possibly caused by autoinflammation with anti-Aβ42 antibodies was shown to be correlated with symptoms of acute or subacute encephalopathy, and is the main MRI finding in CAA-RI. It was suggested that clinical and radiologic criteria may help diagnose probable CAA-RI with good sensitivity and specificity, thereby avoiding cerebral biopsy [3].

In our patient, the response to corticosteroids raised our suspicion of CAA-RI. Initial high-dose methylprednisolone therapy caused rapid improvement of the neurologic symptoms, whereas the sudden cessation of corticosteroid therapy resulted in clinical relapse and deterioration. Subsequent reinitiation of high-dose methylprednisolone followed by slow tapering off of oral prednisone led to clinical and radiological recovery at the 3-month follow-up. The patient’s *ApoE* genotype confirmed our suspicion. According to Antolini et al., 5 IV boluses of high-dose corticosteroids and slow tapering off for several months until clear resolution of acute fluid-attenuated inversion recovery white matter hyperintensities is a reasonable recommendation; there is a higher risk of recurrence during CAA-RI treatment with corticosteroids if IV high-dose IV corticosteroid pulse therapy was suddenly stopped compared to slow tapering off of oral therapy [2]. It should be noted that the patient’s clinical deterioration during the active phase did not meet the definition of recurrence. Kimura et al. also reported a female patient with CAA-RI who received IV methylprednisolone (1000 mg/day for 3 days) without subsequent oral corticosteroids [12]. Her symptoms transiently improved but there was progressive deterioration of consciousness, and mechanical respiratory treatment was required. Therefore, abrupt discontinuation of high-dose corticosteroid without subsequent oral steroids during the active phase of CAA-RI should be avoided.

This report had several limitations. First, we did not test for anti-Aβ autoantibodies in the CSF of our patient, which could increase confidence in discriminating between PRES and CAA-RI and would certainly increase diagnostic accuracy in challenging cases where MRI and clinical data alone are insufficient. Second, biomarkers of neurodegeneration in the CSF including tau and Aβ fragments were not evaluated. Nonetheless, we recommended this analysis to improve management of similar cases in the future.

In conclusion, we described a patient with probable CAA-RI who showed clinical improvement after steroid therapy and was initially thought to have PRES. We suggest that in cases where it is difficult to distinguish between CAA-RI and PRES solely based on MRI, a good response to corticosteroids and *ApoE* ε4/ε4 genotype are critical for establishing a diagnosis of CAA-RI. If clinical deterioration occurs, sudden withdrawal of high-dose corticosteroid during the active phase of CAA-RI should be avoided.

## Supplementary Information

**Additional file 1: Supplementary figure 1.** Head CT on day 10 after withdrawal of the initial methylprednisolone pulse therapy.

## Data Availability

The datasets used and/or analyzed during the current study are available from the corresponding author on reasonable request.

## Abbreviations

CAA-RI: Cerebral amyloid angiopathy related inflammation
CAA: Cerebral amyloid angiopathy
CMBs: Cerebral microbleeds
PRES: Posterior reversible encephalopathy syndrome
ADC: Apparent diffusion coefficient
CSF: Cerebrospinal fluid
BBB: Blood–brain barrier
