# Supplementary figures and images for: Cerebral amyloid angiopathy-related inflammation with posterior reversible encephalopathy syndrome-like presentation: a case report
Source: BMC Neurol. 2022 Dec 3;22:449. doi: 10.1186/s12883-022-02979-6 (PMC9719169; doi:10.1186/s12883-022-02979-6)

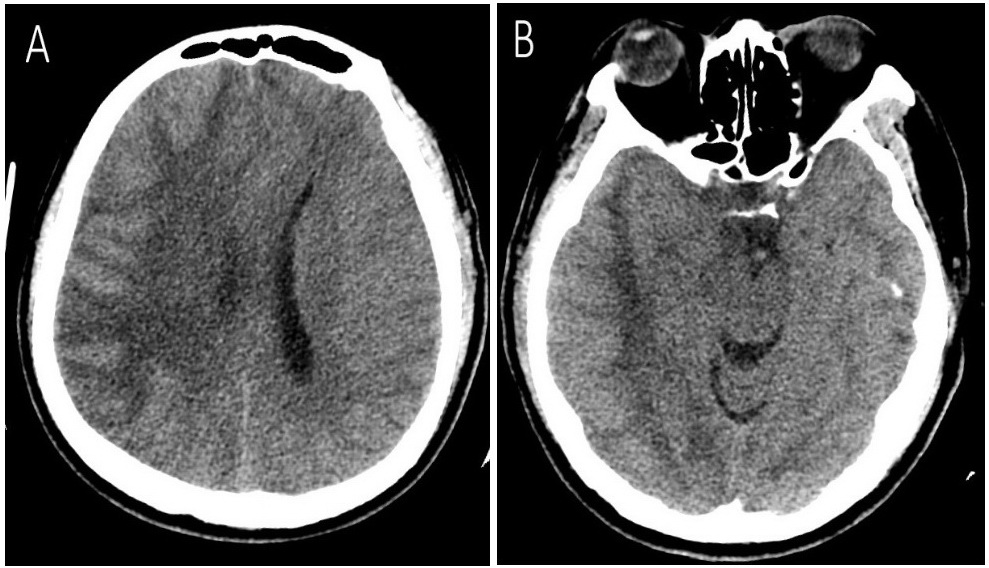

Supplement: Supplementary file 1 — Additional file 1: Supplementary figure 1. Head CT on day 10 after withdrawal of the initial methylprednisolone pulse therapy. [file 12883_2022_2979_MOESM1_ESM.jpg]
